# Cell-type specific distribution and activation of type I IFN pathway molecules at the placental maternal-fetal interface in response to COVID-19 infection

**DOI:** 10.3389/fendo.2022.951388

**Published:** 2023-01-20

**Authors:** Yuping Wang, Yang Gu, David F. Lewis, Xin Gu, Karisa Brown, Courtney Lachute, Miriam Hankins, Rona S. Scott, Caitlin Busada, Danielle B. Cooper, Charles E. McCathran, Perry Barrilleaux

**Affiliations:** ^1^ Department of Obstetrics and Gynecology, Louisiana State University Health Sciences Center - Shreveport, Shreveport, LA, United States; ^2^ Department of Pathology, Louisiana State University Health Sciences Center - Shreveport, Shreveport, LA, United States; ^3^ Department of Immunology and Microbiology, Louisiana State University Health Sciences Center - Shreveport, Shreveport, LA, United States

**Keywords:** COVID-19, STING, IRF3, TLR7, MAVS, type I IFNs, pregnancy, placental maternal-fetal interface

## Abstract

**Background and objective:**

COVID-19 infection in pregnancy significantly increases risks of adverse pregnancy outcomes. However, little is known how the innate immunity at the placental maternal-fetal interface responds to COVID-19 infection. Type I IFN cytokines are recognized as a key component of the innate immune response against viral infection. In this study, we specifically evaluated expression of IFN antiviral signaling molecules in placentas from women infected with COVID-19 during pregnancy.

**Methods:**

Expression of IFN activation signaling pathway molecules, including cyclic GMP–AMP synthase (cGAS), stimulator of interferon genes (STING), interferon regulatory factor 3 (IRF3), Toll-like receptor 7 (TLR7), mitochondrial antiviral-signaling protein (MAVS), and IFNβ were determined in formalin-fixed paraffin embedded (FFPE) placental tissue sections (villous and fetal membrane) by immunostaining. A total of 20 placentas were examined, 12 from COVID-19 patients and 8 from non-COVID-19 controls. Patient demographics, clinical data, and placental pathology report were acquired *via* EPIC medical record review.

**Results:**

Except BMI and placental weight, there was no statistical difference between COVID and non-COVID groups in maternal age, gestational age at delivery, gravity/parity, delivery mode, and newborn gender and weight. In COVID-exposed group, the main pathological characteristics in the placental disc are maternal and fetal vascular malperfusion and chronic inflammation. Compared to non-COVID controls, expression of IFN activation pathway molecules were all upregulated with distinct cell-type specific distribution in COVID-exposed placentas: STING in villous and decidual stromal cells; IRF3 in cytotrophoblasts (CTs) and extra-villous trophoblasts (EVTs); and TLR7 and MAVS in syncytiotrophoblasts (STs), CTs, and EVTs. Upregulation of STING, MAVS and TLR7 was also seen in fetal endothelial cells.

**Conclusions:**

STING, IRF3, TLR7, and MAVS are key viral sensing molecules that regulate type I IFN production. Type I IFNs are potent antiviral cytokines to impair and eradicate viral replication in infected cells. The finding of cell-type specific distribution and activation of these innate antiviral molecules at the placental maternal-fetal interface provide plausible evidence that type I IFN pathway molecules may play critical roles against SARS-CoV-2 infection in the placenta. Our findings also suggest that placental maternal-fetal interface has a well-defined antiviral defense system to protect the developing fetus from SARS-CoV-2 infection.

## Introduction

The pandemic coronavirus disease 2019 (COVID-19) caused by severe acute respiratory syndrome coronavirus 2 (SARS-CoV-2) has significantly impacted public health worldwide. Women infected with COVID-19 during pregnancy results in higher rate of adverse maternal and fetal outcomes, including preterm birth, preeclampsia, stillbirth, gestational diabetes, and low birth weight, than those were not (1–3). Furthermore, emerging studies of placenta, embryos, and cerebral organoids also suggest that fetal organs, such as the brain, could be vulnerable to COVID-19 infection (4). Although newborns delivered to women infected with COVID-19 was reported (5–7), vertical transmission of the virus is rare. However, the findings of detection of SARS-CoV-2 spike protein, nucleocapsid protein, and viral nucleic acids in the placenta from women with COVID-19 infection during pregnancy demonstrated that SARS-CoV-2 can infect placenta (8–11).

Placenta controls oxygen and metabolite exchange, produces growth factors and hormones, and transfers nutrients to support fetal development and growth. Placenta is also an important structural and immunological barrier to prevent pathogen transmission to fetus during pregnancy. In terms of the immune barrier, there are at least 3 boundaries at the maternal-fetal interface: A) intervillous space: where syncytiotrophoblasts (STs) overlay villous tissue and are in direct contact with maternal blood. STs are highly resistant to infection to pathogens (12–14); B) the implantation site or decidua basalis: where the invading extra-villous trophoblasts (EVTs) are in direct contact with maternal decidual cells; and C) fetal membrane, which contains EVTs and maternal decidual cells, is in direct contact with the uterine cavity. Although the placental cells express high levels of antimicrobial defense molecules (15) such as antimicrobial peptides defensins and pattern recognition receptors (PRRs), it remains unclear how the placental defense system functions against SARS-CoV-2 infection at the maternal-fetal interface.

Abnormal vascular development and increased inflammatory response have been characterized in placentas from women infected with COVID-19 during pregnancy as evidenced by substantial maternal vascular malperfusion (MVM) in decidua and fetal vascular malperfusion (FVM) in villous tissue, along with increased focal fibrin deposition and increased lymphocyte and macrophage infiltration, etc. (14–16). These findings indicate that aberrant vascular development and hyper-inflammatory status in the placenta are associated with maternal COVID-19 infection. However, despite fast-tracked intensive research on many aspects of COVID-19, the impact of the viral infection in the placenta and the immune response of the placental defense mechanism(s) are poorly explored.

Interferon (IFN) cytokines are key molecules modulating immune responses and type I IFNs are considered to play crucial roles in protection of pregnancy *via* their antiviral and immune modulatory properties (17–19). The baseline expression of type I IFN is very low in most tissues and could be rapidly triggered by viral attack and bacterial infections. It was reported that SARS-CoV-2 infection in pregnancy is associated with robust inflammatory response at the maternal-fetal interface, with increased activation of natural killer (NK) and T cells and increased expression of interferon-related genes, such as ISG15, an interferon-induced protein (20). In this study, we investigated if type I IFN pathway molecules are differentially activated at the maternal-fetal interface in placentas from women infected with COVID-19 during pregnancy. We specifically evaluated several key molecules that are involved in type I IFN activation, including 1) cyclic GMP–AMP synthase (cGAS); 2) stimulator of interferon genes (STING); 3) interferon regulatory factor 3 (IRF3); The cGAS-STING-IRF3 pathway plays critical roles in the induction of type I IFN activation in cells encountered viral infection (21, 22). We also examined Toll-like receptor 7 (TLR7) and mitochondrial antiviral-signaling protein (MAVS) expression. TLR7 is an endosomal innate immune sensor capable of recognizing single-stranded RNA (ssRNA) of virus infection (23, 24). MAVS is an essential adaptor protein of antiviral immunity in mitochondria, which could activate IRF3 and subsequently induce type I IFN expression (22). We also assessed IFNβ expression, a key type I IFN cytokine. These type I IFN activation markers were examined in both villous tissue and fetal membrane of placentas from women infected with COVID-19 during pregnancy.

## Materials and methods

### Study subjects and placenta specimen

This study was approved by the Institutional Review Board (IRB) at Louisiana State University Health Sciences Center-Shreveport (LSUHSC-S). Formalin-fixed paraffin embedded (FFPE) placental tissue sections, including villous tissue and fetal membrane, were obtained from Pathology archives at LSUHSC-S. A total of 20 placentas were examined, 12 from women infected with COVID-19 during pregnancy and 8 controls from women never infected with COVID-19 before and during pregnancy. Patients diagnosed with COVID-19 infection were detected by polymerase chain reaction (PCR) of SARS-CoV-2 RNA in nasopharyngeal swab specimens. Patients classified as asymptomatic, mild to moderate, or severe were based on their symptoms and clinical findings as defined by the NIH COVID-19 guidelines (25). Demographic and clinical information was obtained by chart review *via* EPIC medical record system. As showed in **Table 1**, BMI was significantly higher in the COVID-19 than in the control group. There were no statistical differences between control and COVID-19 groups in maternal age, gestational age at delivery, gravity/parity, racial status, delivery mode, and newborn gender and weight. However, placental weight was significantly less in the COVID-19 than in the control group.

**Table 1:** Demographic data of pregnant women with or without COVID-19 infection from whom placenta was studied.

Characteristics	Non-COVID Control	COVID-19 Infection	*P* value
	n = 8	n = 12	
Maternal age	29 ± 9	26 ± 7	0.4387
Racial status*, AA/Caucasian	5/3	12/0	0.0491
Gestational age (weeks^+days^)	38^+5^ ± 1^+0^	36^+3^ ± 4^+1^	0.1308
Singleton/Twins*	8/0	11/1	1.0000
Nulliparous*, n (%)	2 (25%)	3 (25%)	1.0000
BMI	31.7 ± 6.5	38.6 ± 6.7	0.0345
Delivery mode*, Vaginaldelivery/C-section	4/4	7/5	1.0000
Placental weight (gram)	615 ± 124	441 ± 134	0.0078
Newborn gender* (male/female)	4/4	7/6	1.0000
Newborn weight (gram)	3405 ± 365	2702 ± 992	0.0711

Data are expressed as mean ± SD. Statistics was calculated by un-paired test; *Statistics on racial status, singleton/twin, nulliparous, delivery mode, and newborn gender were done by Fisher’s exact test. AA, African American; BMI, body mass index.

### Immunohistochemical (IHC) staining

IHC was performed to evaluate expression and distribution of cGAS, STING, IRF3, TLR7, MAVS, and IFNβ in villous and fetal membrane tissue sections from all study subjects. Expression of vimentin (a marker of mesenchymal stromal cells) and CD68 and CD16 (markers of macrophage) were also determined. Villous tissue section contains STs, cytotrophoblasts (CTs), mesenchymal stromal cells (MSCs), Hofbauer cells (placental macrophages), and fetal endothelial cells. Fetal membrane contains amnionic epithelial cells, EVTs, and maternal decidual cells and MSCs. **Supplemental Figure S1** shows a sagittal plan of a placenta and hematoxylin and eosin (H&E) and cytokeratin 5/8 (a marker of trophoblasts) staining in villous and fetal membrane tissue sections, which provide a general overview of villous and fetal membrane structure to show the typical layout and cell distribution of the tissue sections.

A standard immunohistochemistry staining procedure was performed. Briefly, a series of deparaffinization was carried out with xylene and ethanol alcohol. Antigen retrieval was performed by boiling tissue slides with 0.01mol/L citric buffer. Hydrogen peroxide was used to quench the endogenous peroxidase activity. After blocking, tissue sections were incubated with primary antibody overnight at 4°C. Corresponding biotinylate-conjugated secondary antibodies and ABC staining system (Santa Cruz Biotechnology) were used subsequently according to the manufacturer’s instruction. Stained slides were counterstained with Gill’s formulation hematoxylin. Tissue sections stained with isotype IgG or secondary antibody only were used as controls. Stained slides were reviewed under an Olympus microscope (Olympus IX71, Tokyo, Japan). In general, 3-4 images were randomly captured by a digital camera and recorded into a microscope-linked PC computer. The source of antibodies used in the study and antibody dilution factors are present in **Supplemental Table S1**.

For semi-quantification analysis of villous tissue immunostaining, STING and IRF3 positive cells were analyzed using Image J Plugins IHC profiler as described by Seyed Jafari and Hunger (26) with modification. Percentage contribution of high positive and percentage contribution of positive were combined as relative positive cell accounts. The intensity of TLR7, MAVS, and IFNβ staining in villous tissue sections was analyzed using semi-quantitative H-score as described by Lockwood et al. (27). Categories 1-4 were assigned: 1 (negative staining); 2 (detectable but weak staining); 3 (moderate or distinct staining), and 4 (intensive staining). The mean of H-score was generated as relative intensity immunostaining in each specimen. Semi-quantification was not done in fetal membrane staining since cell types and staining intensity can be easily differentiated.

### Statistical analysis

Comparisons of clinical demographic data were performed with un-paired t-test or Chi-square test and data are presented as mean ± SD. Data for villous immunostaining were analyzed with un-paired t-test and expressed as mean ± SE. Computer software Prism 9 (GraphPad Software, Inc. La Jolla, CA) was used. A probability level less than 0.05 was considered statistically significant.

## Results

### Clinical data and placental pathology of COVID-19 subjects

Clinical characteristics of women infected with COVID-19 in pregnancy, including gestational age at COVID-19 infection and delivery, and maternal and newborn complications and outcomes are presented in **Table 2**. There were 2 cases (cases 5 and 6) infected with COVID-19 in the first trimester, 4 cases (cases 7-11) in the second trimester, and 5 cases (cases 1-4 and 12) in the third trimester. Among them, except case 11, who was complicated with antiphospholipid syndrome (APS) and delivered at 26 weeks of gestation, and case 12, a twin pregnancy complicated with severe preeclampsia and asthma, with severe COVID-19 complication of acute respiratory distress syndrome (ARDS) delivered at 30 weeks of gestation, cases 1-10 were all delivered at term or closed to term (**Table 2**). The two preterm cases could impact the relatively lower mean newborn weight in the COVID-19 group. None of the newborns was infected with COVID-19. It was noted that placental weight was significantly less in the COVID group than in the control group, **Table 1**. If excluding cases 11 and 12 (the two preterm births in the COVID group), the mean placental weight was still significantly less in the COVID group than in the control group, 501 ± 77 grams vs. 615 ± 124 grams, p = 0.0289. **Supplemental Figure S2** shows the correlation of newborn weight with placental weight in the control and COVID groups.

**Table 2:** Clinical characteristics of women infected with COVID-19 in pregnancy.

Case#	Maternal Age	Racial Status	Parity	BMI	GA at COVID(+)	GA at Delivery	COVID test at Delivery	COVID-19 Symptoms	Maternal Outcomes	Newborn Outcomes	Newborn COVID Test
1	22	AA	G4P1	36.2	39^+3^	39^+3^	Pos	Asymptomatic	Severe PE	Singleton, M/3260g NICU 3days, Sepsis evaluation	Neg
2	35	AA	G7P5	41.2	37^+0^	39^+1^	Pos	Asymptomatic	Type-2 DM	Singleton, M/3282g, NICU 3days, COVID/glucose monitoring	Neg
3	26	AA	G3P2	36.8	37^+1^	37^+1^	Pos	Mild	Type-2 DM	Singleton, F/3280g NICU 3days, RDS, COVID monitoring	Neg
4	20	AA	G2P0	41.2	35^+4^	38^+1^	NT	Mild	SIPE, CHTN, Syphilis, Marijuana abuse	Singleton, M/3,250g NICU 11days Congenital syphilis	NT
5	20	AA	G1P0	24	8^+3^	40^+0^	Neg	N/A	GBS (+)	Singleton, M/3315g Meconium	NT
6	35	AA	G7P2	41.7	4^+2^	35^+3^	Neg	Mild	PE	Singleton, M/2,500g Extra digit on both hands	NT
7	19	AA	G3P2	30.1	14^+1^	39^+0^	Neg	Mild	GBS (+), Cannabis abuse	Singleton, F/3,670g NICU 1day, Sepsis evaluation	NT
8	39	AA	G8P0	44.5	14^+3^	37^+4^	Neg	Mild	Severe PE, Type I Diabetes	Singleton, F/3,450g	NT
9	20	AA	G1P0	46.5	16^+4^	37^+2^	Neg	N/A	Type-2 DM, Hodgkin Lymphoma	Singleton, F/3,200g	NT
10	33	AA	G6P1	43.8	20^+0^	37^+5^	Neg	Mild	CHTN, GBS(+), Oligohydramnios, Cigarette smoking	Singleton, M/2,770g Abnormal quad screen	NT
11	23	AA	G3P0	33.3	18^+0^	26^+1^	Neg	Asymptomatic	APS, GPS(+), Syphilis, Oligohydramnios	Singleton, F/730g NICU 59 days, Prematurity	NT
12	27	AA	G5P4	43.5	28^+1^	30^+1^	Pos	Severe	ARDS, Severe PE, Asthma, Di/Di Twins, Thrombocytosis, Leukocytosis, Hypokalemia	Twins, M/F. 1,320g/1,100g, Prematurity Sepsis, BPD, RDS, NICU 78/79days	Neg/Neg

AA, African American; GA, gestational age; BMI, body mass index; PE, preeclampsia; SIPE, superimposed preeclampsia; CHTN, chronic hypertension; GBS, Group B Streptococcus; APS, antiphospholipid syndrome; ADRS, acute respiratory distress syndrome; NICU, neonatal intensive care unit; BPD, bronchopulmonary dysplasia; RDS, respiratory distress syndrome. Pos, Positive; Neg, negative; NT, not tested; N/A, information is not available.

**Figure 1:**
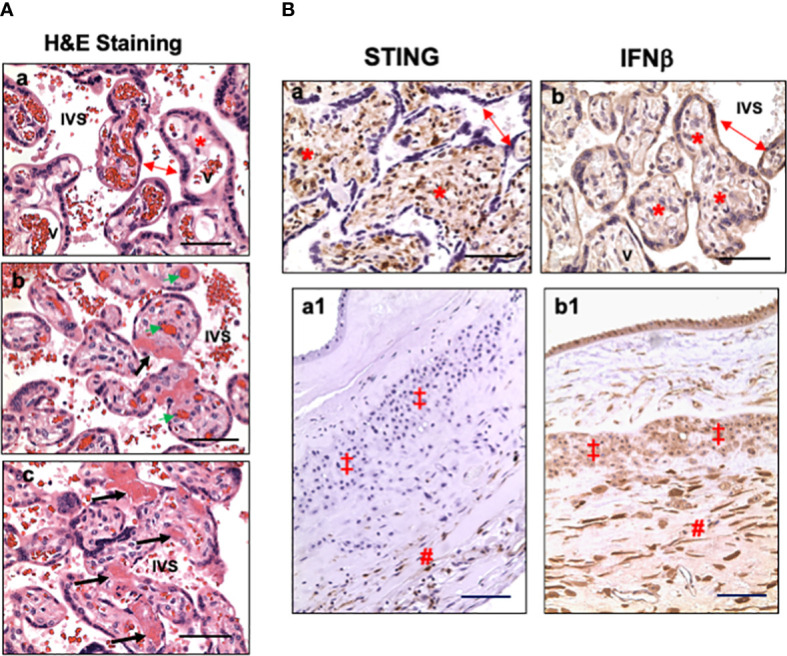
Representative villous tissue H&E staining and expression of STING and IFNβ in villous tissue and fetal membrane in placentas exposed to maternal COVID-19 infection. **(A)** Representative villous tissue H&E staining in COVID-exposed (b and c) placentas compared to non-COVID control (a) showing features of fetal vascular malperfusion including avascular villi, vascular thrombi, and perivascular fibrin deposition in villous tissue from COVID-19 exposed placentas. IVS: intervillous space; double arrow: STs; *: villous stroma; bold arrows: avascular villi and perivascular fibrin deposition; green arrow: fetal vessel thrombi. Bar = 50µm. **(B)** STING and IFNβ expression in villous tissue and fetal membrane in placentas exposed to maternal COVID-19 infection. a and b: villous tissue; a1 and b1: fetal membrane; a and a1: STING; and b and b1: IFNβ. In villous tissue: double arrow: STs; *: villous stroma. In fetal membrane: ‡: EVTs; #: decidua. Bar = 50µm in a and b; Bar = 100µm in a1 and b1.

Characteristics of placental microscopic pathology for COVID-19 patients were extracted from pathology report, re-evaluated by pathologist at LSUHSC-S, and presented in **Table 3**. The main pathological characteristics in placental disc are maternal and fetal vascular malperfusion and chronic inflammation. The main features of maternal vascular malperfusion include infarct, intervillous thrombus, and peri-villous fibrin deposition. The main features of fetal vascular malperfusion include avascular villi, vascular thrombi (infarction), and perivascular fibrin deposition. Chronic deciduitis is the major inflammatory pathology highlighted in the placental disc. For fetal membrane, 6 out of the 12 cases showed acute or chronic chorioamnionitis and/or chronic deciduitis. These placental vascular abnormalities and inflammatory marks found in our study are consistent with what were reported previously (8, 11, 13, 14). **Figure 1A** shows representative H&E staining in control (a) and COVID placentas (b and c). H&E staining in COVID-exposed placentas clearly shows the features of fetal vascular malperfusion including avascular villi, vascular thrombi, and perivascular fibrin deposition in villous tissue from COVID-19 exposed placentas.

**Table 3:** Placental microscopic pathology of COVID-19 subjects.

Case# Placenta	Weight (grams)	Placental Disc	Fetal Membrane
1	556	intervillous thrombus, infarction, dystrophic calcification	acute chorioamnionitis
2	538	focal infarct, subchorionic hematoma	no histopathological abnormality
3	451	peri-villous fibrin deposition	no significant pathology finding
4	570	fibrin deposition, villous congestion	acute chorionitis
5	430	fibrin deposition	necrotic changes
6	394	fibrin deposition, focal infarction	no significant pathology finding
7	568	fibrin deposition, calcification, focal chorangiomatosis	chorioamnionitis
8	487	focal infarction, fibrin deposition	no significant pathology finding
9	409	decidual vasculopathy	acute chorioamnionitis
10	608	villous congestion, subchorionic hematoma	no histopathological abnormality
11	194	acute chorioamnionitis chorionic plate vasculitis	acute chorioamnionitis
12	526* dichorionic, diamnionic twins	A: lymphoplasmacytic infiltration chronic deciduitis B: lymphoplasmacytic infiltration chronic deciduitis	Chronic deciduitis lymphoplascytic infiltration chronic deciduitis lymphoplasmacytic infiltration

*Total placental weight.

### Detection of STING and IFNβ expression in villous tissue and fetal membrane

To determine if type I IFN pathway is activated in placentas exposed to maternal COVID-19 infection, we first examined cGAS, STING, and IFNβ expression. Our results showed that cGAS expression was neither detected in villous nor fetal membrane tissue sections in placentas with or without exposure to COVID-19 in pregnancy (data not shown). Surprisingly, abundant STING positive cells were detected in villous stroma in all placentas in the COVID group (**Figure 1B**, a). Stromal cells in chorionic mesoderm (CM) and decidual layer in fetal membrane also showed positive STING staining (**Figure 1B**, a1). STING expression was not detected in trophoblasts, including STs, CTs, and EVTs, in COVID-19-exposed placentas. Interestingly, strong IFNβ expression was detected in villous trophoblasts (STs, CTs) (**Figure 1B**, b) in COVID-19-exposed placentas. IFNβ signal was also detected in fetal membrane cells, including amnion epithelial cells, EVTs, stromal cells in chorionic mesoderm (CM) and decidual layer (**Figure 1B**, b1). These results suggest that STING and IFNβ expressing cells are cell type-specific at the placental maternal-fetal interface in response to maternal COVID-19 infection.

**Figure 2:**
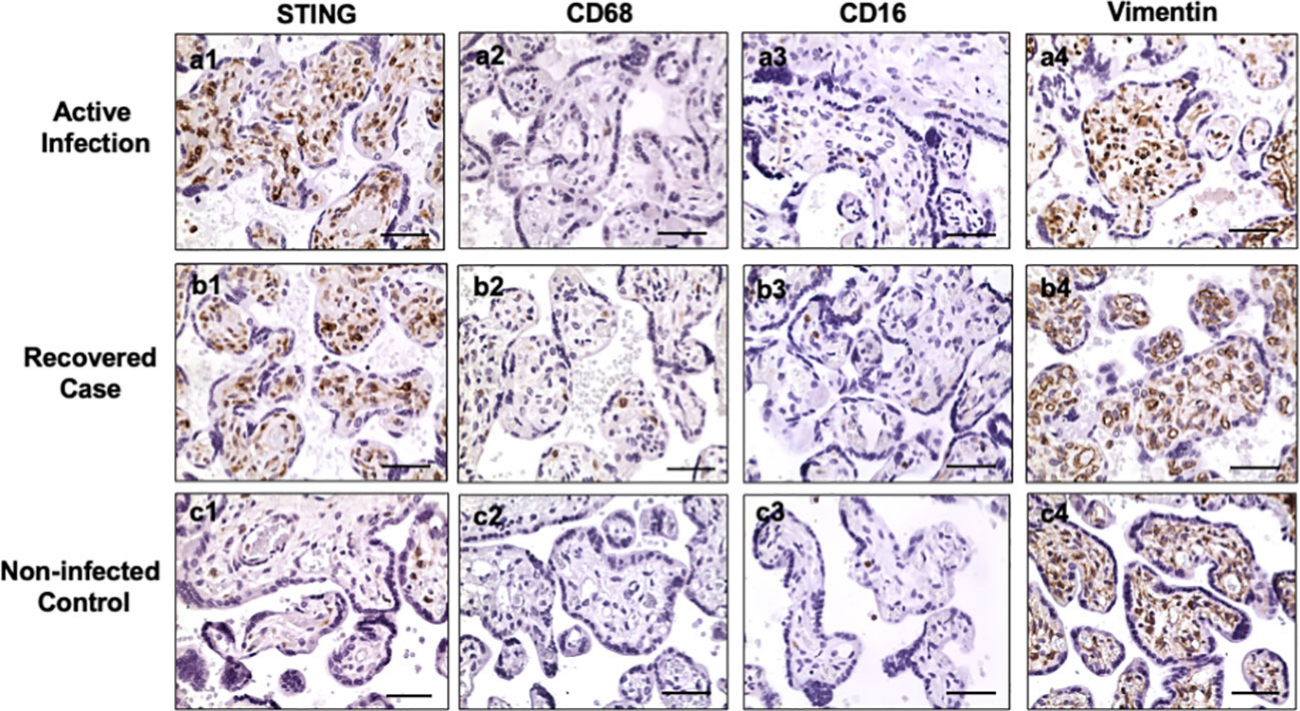
STING, CD68, CD16, and vimentin expression in villous tissue of placentas with or without exposure to maternal COVID-19 infection. Active infection: from placentas delivered by women with positive detection of SARS-COV-2 RNA when admitted to Labor and Delivery. Recovered case: from placentas delivered by women with positive detection of SARS-COV-2 RNA at second trimester and negative detection of SARS-COV-2 RNA when the patient was admitted to hospital for delivery. Non-infected control: from placenta delivered by women who was never infected with COVID-19 before and during pregnancy. STING is strongly expressed in villous stromal cells in both active infection (a1) and recovered (b1) cases, but only a few positive cells were seen in villous stromal in non-infected control (c1) placentas. Both CD68 and CD16 are markers for macrophages. Only a few positive CD68 and CD16 cells were detected in villous stromal in active (a2, a3), recovered (b2, b3), and control (c2, c3) placentas. Vimentin is a marker of mesenchymal cells. Vimentin positive cells were detected in villous stroma in all villous tissue examined: active infection (a4), recovered (b4), and non-COVID control (c4) placentas. These results indicate that STING positive cells are villous mesenchymal stromal cells (MSCs), not Hofbauer cells (macrophages), in placentas exposed to COVID infection. Bar = 50µm.

Hofbauer cells (placental macrophages) locate in villous stroma. To determine if the STING positive cells are Hofbauer cells, expression of CD68, CD16, and vimentin were determined in villous tissue sections. CD68 and CD16 are markers of macrophages, and vimentin is a marker of stromal mesenchymal cells. **Figure 2** shows representative STING, CD68, CD16, and vimentin expression in villous tissue sections from active infection and COVID-19 recovered cases, and non-infected controls. Active infection was the placenta from pregnant women with positive detection of SARS-CoV-2 RNA in nasopharyngeal swab specimen when admitted to hospital for delivery. COVID-19 recovered was the placenta from women infected with COVID-19 during first or second trimester with negative detection of SARS-CoV-2 RNA when admitted to hospital for delivery. Non-infected control was a placenta from women never infected with COVID-19 before and during pregnancy. Abundant STING expressing cells are seen in villous stroma in placentas from COVID-19 infected cases (**Figure 2**, a1, b1), but not in non-COVID controls (**Figure 2**, c1). For CD68 and CD16 expression, only a few positive cells were detected in villous tissue sections from COVID-19-exposed and non-COVID placentas, CD68: **Figure 2**, a2, b2, and c2 and CD16: a3, b3, and c3, respectively. However, abundant vimentin positive cells were detected in villous stroma in all specimens regardless of COVID-19 infection status, **Figure 2**, a4, b4, and c4. These results demonstrate that STING positive cells may not be macrophages, but MSCs. These results also suggest that placental villous MSCs are activated in response to maternal COVID-19 infection.

### Differential activation of type I IFN pathway molecules in villous tissue cells in placentas from women infected with COVID-19 in pregnancy

**Figure 3:**
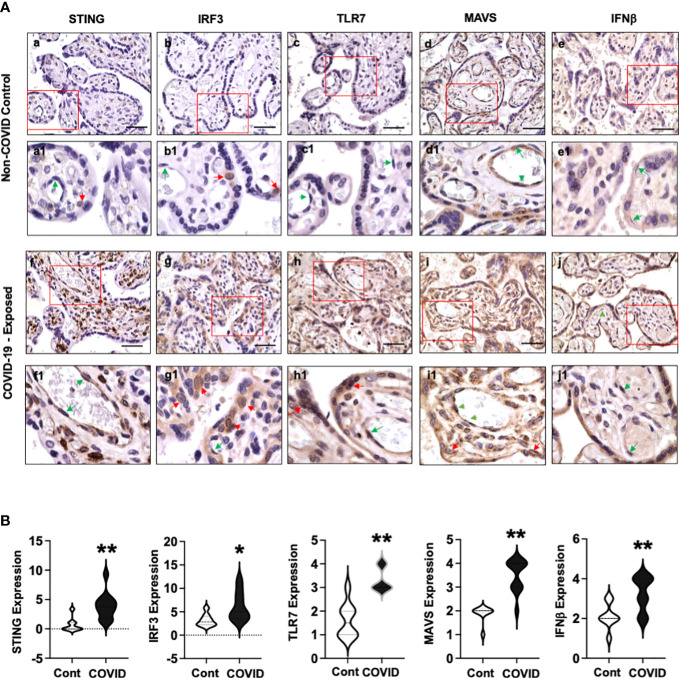
STING, IRF3, TLR7, MAVS, and IFNβ expression in villous tissue of placentas with or without exposure to maternal COVID-19 infection. **(A)** STING, IRF3, TLR7, MAVS, and IFNβ expression in villous tissues from placentas with or without exposure to maternal COVID-19 infection. Images a to e are representative images of STING, IRF3, TLR7, MAVS, and IFNβ expression in villous tissue sections in non-COVID control placentas, and images a1 to e1 show zoom of enlarged rectangle area in a to e of each, respectively. Images f to j are representative images of STING, IRF3, TLR7, MAVS, and IFNβ expression in villous tissue sections in COVID-exposed placentas, and images f1 to j1 show zoom of enlarged rectangle area in f to j of each, respectively. Bar = 50µm. Activation or upregulation of STING, IRF3, TLR7, MAVS, and IFNβ expression were found in different cell-types in COVID-exposed placental villous tissue compared to non-COVID controls: 1) strong STING expression signal in stromal cells and fetal endothelial cells; 2) activation of IRF3 in CTs; and 3) increased TLR7 expression in STs, CTs, and fetal endothelial cells; 4) upregulation of MAVS expression in STs, CTs, stromal cells, and fetal endothelial cells; and 5) increased IFNβ expression in STCs and fetal endothelial cells, respectively. Red arrow: CTs; Green arrow: fetal endothelial cells. **(B)** Violin graphs show that relative expression for STING, IRF3, TLR7, MAVS, and IFNβ in villous tissue sections are significantly increased in COVID-exposed placental villous tissue (COVID) vs. non-COVID controls (Cont), * p<0.05 and ** p<0.01, respectively.

**Figure 3A** shows representative images of STING, IRF3, TLR7, MAVS, and IFNβ expression in villous tissue sections from COVID-19 exposed placentas compared to non-COVID controls. Again, abundant STING positive cells were detected in stromal cells and fetal endothelial cells in COVID-19 exposed placentas (**Figure 3A**, f and f1) vs. non-COVID controls (**Figure 3A**, a and a1). Interestingly, increased IRF3 expression was detected in CTs in COVID-19 exposed placentas (**Figure 3A**, g and g1) compared to that in non-COVID controls (**Figure 3A**, b and b1). STING and IRF3 expression were not detected in STs in COVID-19 exposed placentas. In contrast to STING and IRF3 expression, upregulation of TLR7 was seen in both STs, and CTs in COVID-19 exposed placentas (**Figure 3A**, h and h1) compared to non-COVID controls (**Figure 3A**, c and c1). Upregulation of TLR7 expression was also seen in stromal cells and fetal endothelial cells in COVID-19 exposed placentas (**Figure 3A**, h and h1). STING, IRF3, and TLR7 are all viral sensing molecules in type I IFN pathway. The findings of STING, IRF3, and TLR7 upregulation in different cell-types in villous tissue of COVID-19 exposed placentas suggest that cells at the placental maternal-fetal interface react differently in response to maternal COVID-19 infection.

MAVS plays a major role in antiviral defense mechanisms by coordinating and activating IFN pathway signaling. Our results showed that MAVS was expressed in STs and fetal endothelial cells in villous tissue of non-COVID control placentas (**Figure 3A**, d and d1). However, MAVS expression was robustly upregulated in all villous cells, including STs, CTs, stromal cells, and fetal endothelial cells in COVID-exposed placentas (**Figure 3A**, i and i1) compared to the controls (**Figure 3A**, d and d1). The observation of MAVS upregulation in all villous cells provides further evidence that mitochondria act as a platform and are actively involved in antiviral immunity against SARS-CoV-2 infection in cells at the maternal-fetal interface. Compared to non-COVID controls (**Figure 3**, e and e1), strong IFNβ expression was detected in placental STs and fetal endothelial cells in villous tissue in COVID-19 exposed placentas (**Figure 3**, j and j1), respectively.

**Figure 3B** shows relative expression of STING, IRF3, TLR7, MAVS, and IFNβ in villous sections from COVID-19 exposed placentas compared to non-COVID controls. Significant upregulation of STING, IRF3, TLR7, MAVS, and IFNβ expression was found in COVID-19 exposed placentas vs. controls (p<0.01 for STING, TLR7, MAVS, and IFNβ, and p<0.05 for IRF3).

### Differential activation of type I IFN pathway molecules in fetal membrane cells in placentas from women infected with COVID-19 in pregnancy

**Figure 4:**
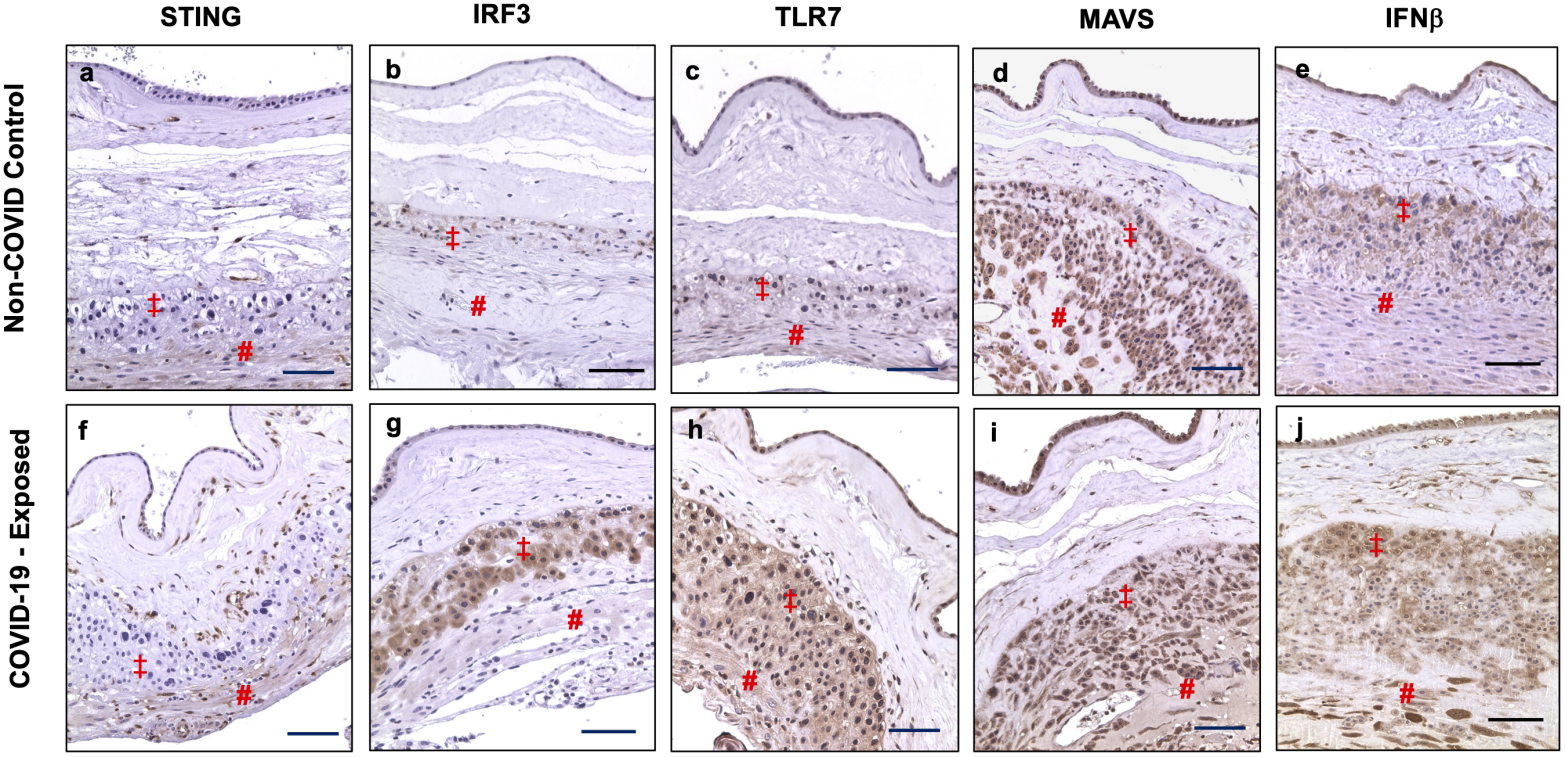
STING, IRF3, TLR7, MAVS, and IFNβ expression in fetal membrane of placentas with or without exposure to maternal COVID-19 infection. Images **a–e** show STING, IRF3, TLR7, MAVS, and IFNβ expression in fetal membrane from non-COVID control placentas, and images **f–j** show STING, IRF3, TLR7, MAVS, and IFNβ expression in fetal membrane from COVID-exposed placentas. Upregulation of IRF3 in EVTs, and increased STING, TLR7, and IFNβ expression in chorionic mesoderm and decidual stromal cells were detected in fetal membrane of COVID-exposed vs. non-COVID control placentas. Increased IFNβ expression was also noticed in EVTs in fetal membrane of COVID-exposed placentas. ‡: EVTs and # decidual cells. Bar = 100µm.

We also examined STING, IRF3, TLR7, MAVS, and IFNβ expression in fetal membrane from non-COVID and COVID-exposed placentas. We found that differential upregulation of these antiviral molecules in fetal membrane cells are also present in COVID-exposed placentas. **Figure 4** shows representative images of STING, IRF3, TLR7, MAVS, and IFNβ expression in non-COVID controls (**Figure 4**, a to e) and in COVID-exposed (**Figure 4**, f to j) fetal membranes, respectively. Compared to non-COVID controls, increased STING expression in chorionic mesoderm and decidual stromal cells, and upregulation of IRF3 in EVTs were observed in fetal membranes in COVID-exposed placentas. TLR7 expression was also upregulated in EVTs and decidual cells in COVID-exposed placentas. Although, MAVS expression did not show different in fetal membrane cells between non-COVID control and COVID-exposed placentas, upregulation of IFNβ expression was detected in EVTs and decidual cells in COVID-exposed in comparison to non-COVID control placentas.

## Discussion

In the present study, we specifically examined expression of antiviral sensing molecules associated with activation of type I IFNs in placentas with or without maternal COVID-19 infection. Strikingly, we found that compared to non-COVID controls, antiviral sensing molecules including STING, IRF3, and TLR7 that link to type I IFN production are substantially upregulated with distinct cell type specific distribution at the maternal-fetal interface in COVID-exposed placentas, i.e., STING in villous and decidual MSCs; IRF3 in CTs and EVTs; and TLR7 in almost all three types of trophoblasts, fetal vessel endothelial cells, and decidual stromal cells.

STING activation in villous MSCs in COVID-19-exposed placentas is a novel finding in our study. Demonstration that STING expressing cells are MSCs, but not Hofbauer cells, is supported by the pattern of vimentin expressing cells in the villous stroma with no differences in CD68 and CD16 positive cells in both COVID-19 infected and non-infected placentas. There are number of biomarkers for macrophages, including CD14, CD16, CD64, CD68, CD71, and CD163, etc. Using CD68 as the marker for placental Hofbauer cells, Rebutini et al. studied the correlation of COVID-19 severity in pregnant women with placental morphologic features. Their results showed no difference in CD68 account in placentas between control and COVID-19 cases (13), which was consistent with ours. While using CD163 as the marker for macrophages, Sharps et al. did find increased CD163 positive cells in villous tissue following maternal COVID-19 infection (16). Increased placental macrophages was also reported in severe preeclampsia complicated by HELLP syndrome (28). It is possible that different subset of macrophages might be activated in the placenta in response to maternal COVID-19 infection and in placentas from various pregnancy complications, which warrant further investigation.

STING is an endoplasmic reticulum-associated membrane protein and can be activated by cGAS when cGAS recognizes cellular DNA in cytosol. cGAS-STING pathway plays critical roles in inducing type I IFN production and activating innate immune defense system in response to both DNA and RNA viral infection (21). Our results of undetectable cGAS expression in villous tissue and fetal membrane from COVID-19 placentas suggest that STING activation in villous and decidual stromal cells is cGAS-independent. In fact, cGAS-independent STING activation has been reported and considered as non-canonical activation of STING in cells after DNA damage (29). Stromal cells possess diverse immune regulatory capacities (30). Although villous stromal cells do not directly contact to maternal components that circulate in placental intervillous space, the finding of STING activation in villous stromal cells suggests that these cells are activated in response to maternal SARS-CoV-2 infection, which could be a feature of antiviral defense network at the placental maternal-fetal interface.

Another important finding is increased IRF3 levels in villous CTs and chorion EVTs in COVID-19 exposed placentas. IRF3 is a member of the interferon regulatory transcription factor (IRF) family. IRF3 activation could lead to type I IFN production in cells respond to viral infection (31). EVTs or invasive trophoblasts are trophoblasts residing outside of villi, which can be found in chorion, cell columns, basal plate, and decidua basalis. These cells directly contact maternal cells, including decidual cells, macrophages, dendritic cells, and T cells, etc. Currently, little is known about specific immune function of EVTs in viral infection, the finding of IRF3 activation in chorion EVTs suggests that EVTs may play vital roles at the boundary of EVTs and maternal decidual cells against COVID infection. Further study is needed to define the function of EVTs in the host innate immune system.

Elevated TLR7 positivity was noticed almost in all types of cells in villous tissue and fetal membrane, including STs, CTs, EVTs, fetal endothelial cells, amnion epithelial cells, and maternal decidual cells. It is known that among the TLR family members, TLR7 and TLR8 are recognized as sensors to ssRNA viruses (32) and SARS-CoV-2 genome contains a large number of fragments that can be recognized by TLR7/8 (24). TLR7/8 are X-linked genes. It was reported that X-linked recessive TLR7 deficiency was a highly penetrant genetic etiology of critical COVID-19 pneumonia (33, 34) and deficient TLR7 gene was associated with severity of SARS-CoV-2 infection in young male patients (35). Further study needs to determine if TLR7 gene deficiency in placenta or fetus is associated with vertical transmission of SARS-CoV-2 in pregnancy. It is worth to note that upregulation of TLR7 expression was also observed in villous core fetal endothelial cells in COVID-exposed placentas. Activation of TLR receptors has been linked to endothelial dysfunction in various cardiovascular diseases, such as atherosclerosis, hypertension, and ischemic injury (36). Although endothelial cells are not considered as classical immune cells, they are actively involved in inflammatory responses to various stimuli. Increased TLR7 expression seen in fetal endothelial cells could be a sign of fetal vascular response to maternal COVID infection. Whether TLR7 upregulation contributes to fetal endothelial cell dysfunction warrants further investigation. Nonetheless, upregulation of TLR7 expression in cells at the maternal-fetal interface indicates that TLR7 plays an important role in response to maternal COVID-19 infection.

MAVS is an adaptor protein that locates in multiple intracellular membranous compartments including mitochondria, peroxisomes, and endoplasmic reticulum (37). MAVS is also known as IFN-β promoter stimulator I (IPS-1) or virus induced signaling adaptor (VISA) (38). It is activated when pattern recognition receptors, such as retinoic acid-inducible gene I (RIG-I)-like receptors and melanoma differentiation-associated gene 5 (MDA5), detect the presence of viruses within cells. Aggregated MAVS activates a series of cellular responses which directly induce type I IFN production. Upregulation of MAVS expression in villous STs in COVID-exposed placentas indicates that MAVS could be a key antiviral sensing molecule in STs. Moreover, the finding of strong MAVS signals in fetal membrane cells in both control and COVID-exposed placentas also suggests that MAVS may play a dominant antiviral role in EVTs at the maternal decidual boundary.

Increased STING, IRF3, TLR7, and MAVS expression directly link to IFN activation in cells at the maternal-fetal interface in COVID-exposed placentas. This notion is supported by the finding of strong IFNβ (a type I IFN) expression in villous STs and in EVTs and maternal decidual cells in fetal membrane in COVID-exposed placentas. IFN response is considered a first line of defense against viral infection because it promotes virus clearance, induces tissue repair, and triggers a prolonged adaptive immune response against viruses (39). Type I IFN family has several members, including IFNα, IFNβ, IFNε, IFNω, and IFNv. IFNα and IFNβ are major type I IFNs. Humans produce 13 IFNα and 2 IFNβ. We evaluated IFNβ1 expression, and our results showed that IFNβ1 expression was robustly upregulated especially in STs and maternal decidual cells in COVID-exposed placentas, which indicates activation of IFNβ1 in cells at the maternal-fetal interface. IFN signaling is a main driver of the antiviral defense. Therefore, our findings support the notion that activation of type I IFN signaling pathway is a pivotal antiviral defense mechanism at the maternal-fetal interface against SARS-CoV-2 infection.

In addition, we did notice that placental weight was significantly less in the COVID group than in the control group (**Table 1**). Our data also showed that newborn weight was correlated with placental weight (**Supplemental Figure 2**). As mentioned early that abnormal vascular development and hyper-inflammatory status in the placenta are associated with maternal COVID-19 infection. There is no doubt that COVID-19 infection during pregnancy has significant impact on placental vascular development, which may account to low birth weight in COVID cases. Whether type I IFN upregulation plays a role in aberrant placental vasculature development warrants further investigation.

There are some limitations in our study. This is a retrospective specimen analysis using archived placental tissues. All placentas in the COVID-19 group are from Africa American women. This disproportionate COVID-19 infection population represents the demographic and ethnic disparities in the Shreveport community. Although the sample size is small and the intensity of immunostaining in fetal membrane is not quantified, the staining results are consistent showing distinct cell-type specific upregulation of the markers tested in the COVID group. Since SARS-CoV-2 antibody test was not the standard of care and no maternal and fetal blood specimen were preserved, viral load and maternal/fetal antibody (IgG and IgM) levels were not available to these subjects. Therefore, whether activation of antiviral IFN signaling pathway at the maternal-fetal interface correlates with maternal antibody levels or transplacental antibody transfer are not known. It is also unknown if maternal type I IFN levels affect IFN signaling pathway activation at the maternal-fetal interface in placentas exposed to CIVID-19 infection.

## Conclusion

**Figure 5:**
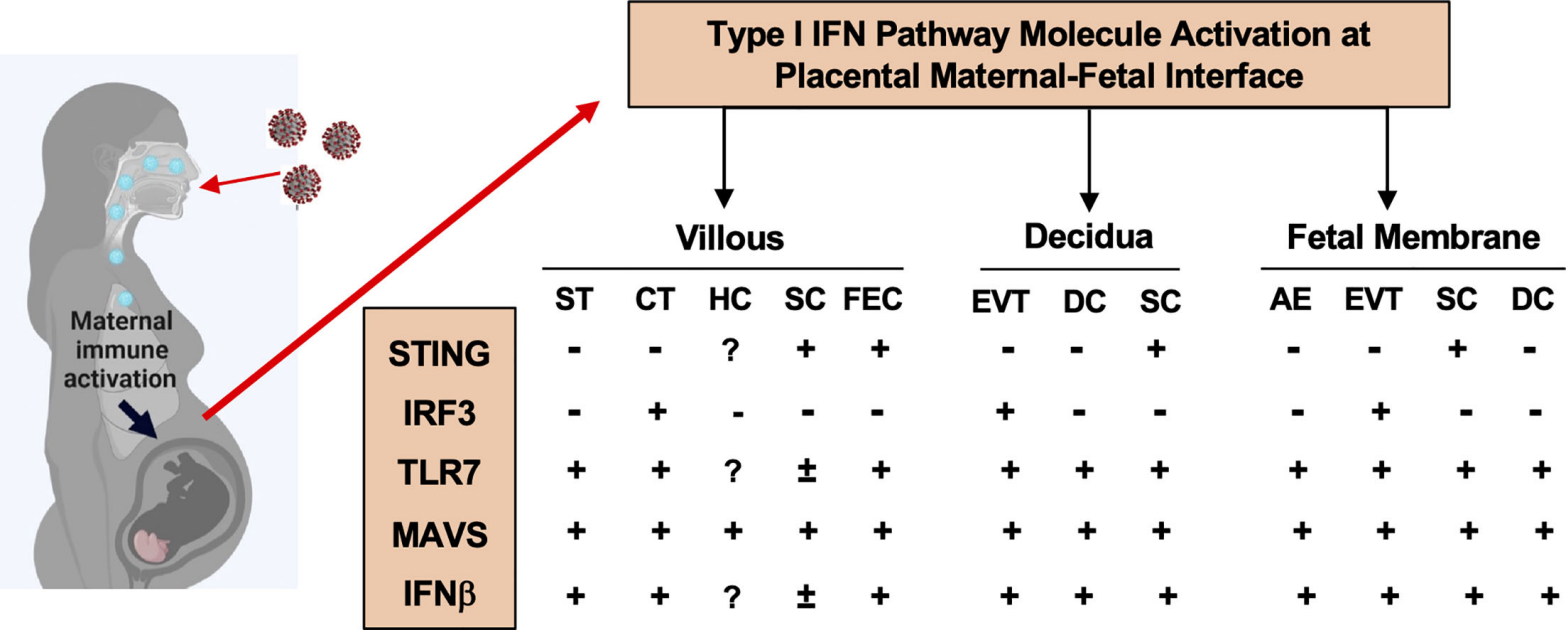
A summary of type I IFN pathway molecule activation in different cell types at the maternal-fetal interface in placentas from women infected with COVID-19 in pregnancy. ST, Syncytiotrophoblasts; CT, cytotrophoblasts; HC, Hofbauer cells; SC, stromal cells; FEC, fetal endothelial cells; DC, decidual cells; AE, amnion epithelial cells; EVT, extra-villous trophoblasts, respectively.

In the present study, we identified distinct cell-type specific sensing and activation of type I IFN pathway molecules at the maternal-fetal interface in placentas exposed to maternal COVID-19 infection. As summarized in **Figure 5**, STING in villous and decidual MSCs; IRF3 in CTs and EVTs; and MAVS, and TLR7 in STs. Upregulation of MAVS and TLR7 was also seen in fetal endothelial cells. These findings highlight that type I IFN signaling pathway is an important antiviral defense network at the maternal-fetal interface in the course of SARS-CoV-2 infection in pregnancy. Our data also suggest that the placenta has a well-defined innate antiviral defense system to limit and eradicate SARS-CoV-2 infection, which could, in part, explain why vertical transmission of SARS-CoV-2 is rare in pregnancy.

## Data availability statement

The original contributions presented in the study are included in the article/**Supplementary Material**. Further inquiries can be directed to the corresponding author.

## Ethics statement

The studies involving human participants were reviewed and approved by Institutional Review Board (IRB) at Louisiana State University Health Sciences Center - Shreveport approved the study entitled “Effects of SARS-CoV-2 infection in women during pregnancy on maternal and fetal outcomes and placental pathology” on 04/30/2021. The IRB ID number is STUDY00001744. Written informed consent for participation was not required for this study in accordance with the national legislation and the institutional requirements.

## Author contributions

YW, YG, DL, XG: study design. YW, YG, XG, KB, CL, MH, CB, DC: specimen process, analysis, data interpretation. YW, DL, RS, CM, PB: drafting and revising manuscript. All authors contributed to the article and approved the submitted version.
