# Supplementary material for: Cell-type specific distribution and activation of type I IFN pathway molecules at the placental maternal-fetal interface in response to COVID-19 infection
Source: Front Endocrinol (Lausanne). 2023 Jan 20;13:951388. doi: 10.3389/fendo.2022.951388 (PMC9895786; doi:10.3389/fendo.2022.951388)
Supplement: Supplementary file 1 [file Table_1.pdf]

Cell-type specific distribution and activation of type I IFN pathway molecules at the placental maternal-fetal interface in response to COVID-19 infection

Yuping Wang, Yang Gu, David F. Lewis, Xin Gu, Karisa Brown, Courtney Lachute, Miriam Hankins, Rona S. Scott, Caitlin Busada, Danielle B. Cooper, Charles E. McCathran, Perry Barrilleaux

Supplementation Table 1: List of antibodies used in the study

Name	Catalog #	Antibody Dilution	Sources
cGAS	79978, 15102	1:50	Cell Signaling (Danvers, MA)
STING	13647	1:100	Cell Signaling (Danvers, MA)
IRF3	10949	1:100	Cell Signaling (Danvers, MA)
TLR7	NBP2-24906	1:200	Novus Biologicals (Centennial, CO)
MAVS	14341-1-AP	1:200	Proteintech (Rosemont, IL)
IFNβ1	BP1-77288	1:200	Novus Biologicals (Centennial, CO)
Cytokeratin 5/8	sc-8021	1:200	Santa Cruz (Santa Cruz, CA)
Vimentin	sc-6260	1:200	Santa Cruz (Santa Cruz, CA)
CD16	24326	1:100	Cell Signaling (Danvers, MA)
CD68	sc-17832	1:50	Santa Cruz (Santa Cruz, CA)
Biotinylated goat anti-rabbit IgG (H+L)	BA-1000	1:200	Vector Laboratories (Burlingame, CA)
Biotinylated goat anti-mouse IgG (H+L)	BA-9200	1:200	Vector Laboratories (Burlingame, CA)
