# Supplementary material for: Cell-type specific distribution and activation of type I IFN pathway molecules at the placental maternal-fetal interface in response to COVID-19 infection
Source: Front Endocrinol (Lausanne). 2023 Jan 20;13:951388. doi: 10.3389/fendo.2022.951388 (PMC9895786; doi:10.3389/fendo.2022.951388)
Supplement: Supplementary file 2 [file DataSheet_1.pdf]

Cell-type specific distribution and activation of type I IFN pathway molecules at the placental maternal-fetal interface in response to COVID-19 infection

Yuping Wang, Yang Gu, David F. Lewis, Xin Gu, Karisa Brown, Courtney Lachute, Miriam Hankins, Rona S. Scott, Caitlin Busada, Danielle B. Cooper, Charles E. McCathran, Perry Barrilleaux

Supplemental Figure 1

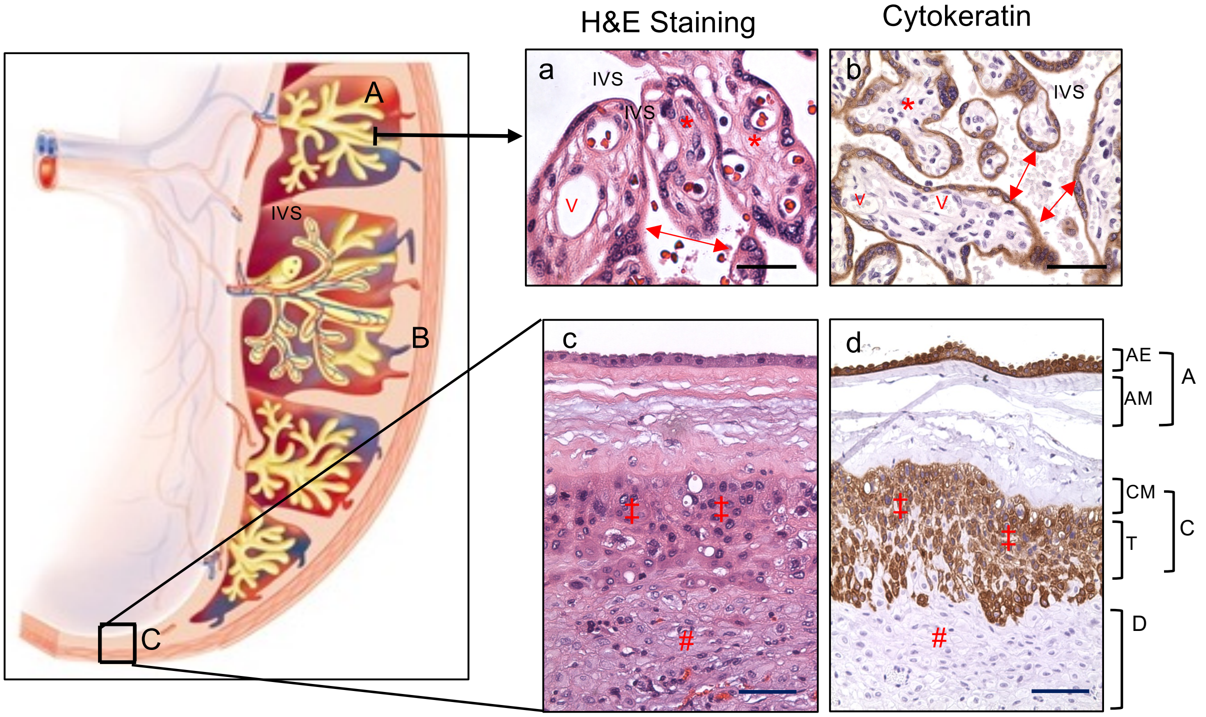

Suppl Figure 1. An illustration of placental maternal-fetal interface.

Left panel: a sagittal plan of a placenta with the cord. A: villous tissue and intervillous space; B: the implantation site or decidua basalis; and C: fetal membrane.

Right panel: Hematoxylin and eosin (H&E) staining in villous (a) and fetal membrane tissue (c) sections and cytokeratin 5/8 (a marker of epithelial cells and trophoblasts) staining in villous (b) and fetal membrane (d) tissue sections which provide a general overview of villous and fetal membrane structure to show the typical layout and cell distribution of the tissue sections.

In a and b: double arrow: syncytiotrophoblasts (STCs); *: villous stroma; v: fetal vessel.

In c and d: ‡: extra-villous trophoblasts (EVTs); #: decidua.

In d: A: amnion; AE: amnion epithelium; AM: amnionic mesoderm; C: Chorion; CM: chorionic mesoderm; T: EVTs; D: decidua. Bar = 50 micon in a and b; Bar = 100 micron in c and d.

Cell-type specific distribution and activation of type I IFN pathway molecules at the placental maternal-fetal interface in response to COVID-19 infection

Yuping Wang, Yang Gu, David F. Lewis, Xin Gu, Karisa Brown, Courtney Lachute, Miriam Hankins, Rona S. Scott, Caitlin Busada, Danielle B. Cooper, Charles E. McCathran, Perry Barrilleaux

Supplemental Figure 2

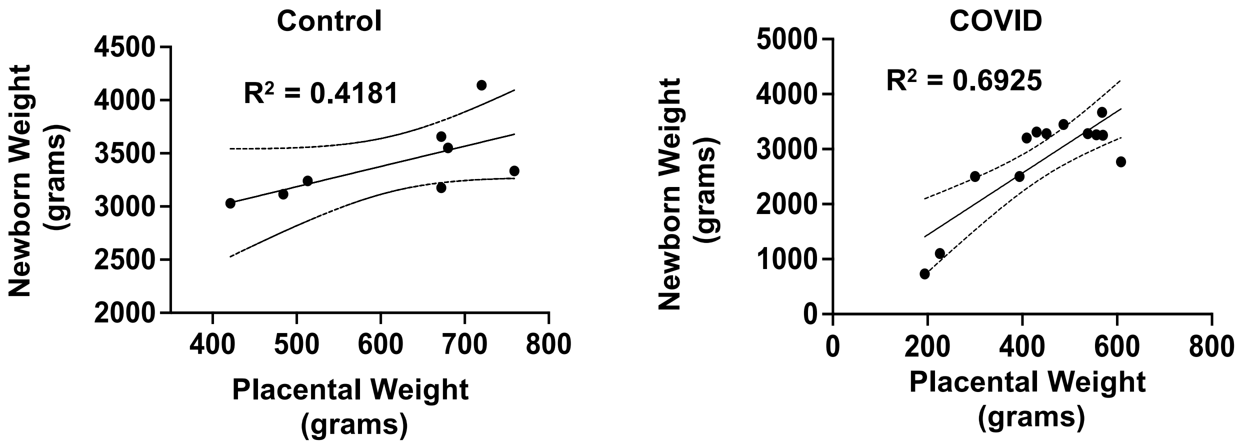

Suppl Figure 2. Correlation of newborn weight with placental weight in non-COVID and COVID groups.
